# Regulation of SOX9 in normal and osteoarthritic equine articular chondrocytes by hyperosmotic loading

**DOI:** 10.1016/j.joca.2010.08.011

**Published:** 2010-11

**Authors:** M.J. Peffers, P.I. Milner, S.R. Tew, P.D. Clegg

**Affiliations:** Dept. of Musculoskeletal Biology, Institute of Ageing and Chronic Disease, University of Liverpool, Leahurst, Neston, Wirral , CH64 7TE, UK

**Keywords:** Cartilage, SOX9, ERK signalling, Osmotic loading

## Abstract

**Objectives:**

SOX9 is a transcription factor that is essential for cartilage extracellular matrix (ECM) formation. Osteoarthritis (OA) is characterised by a loss of cartilage ECM. In chondrocytes SOX9 gene expression is regulated by osmotic loading. Here we characterise SOX9 mRNA regulation through static and cyclical application of hyperosmotic conditions in normal and OA monolayer equine chondrocytes. Furthermore, we investigate whether extracellular signal-regulated protein kinase (ERK)1/2 mitogen-activated protein kinases (MAPK) pathways have a role in this regulation of SOX9.

**Methods:**

Equine chondrocytes harvested from normal or OA joints were subjected to different osmotic loading patterns as either primary (P0) or passaged (P2) cells. The involvement of MEK–ERK signalling was demonstrated by using pharmacological inhibitors. In addition SOX9 gene stability was determined. Levels of transcripts encoding SOX9, Col2A1 and aggrecan were measured using qRT-PCR. *De novo* glycosaminoglycan synthesis of explants was determined with ^35^S sulphate during static hyperosmolar loading.

**Results:**

MEK–ERK signalling increases glycosaminoglycans (GAG) synthesis in explants. Static hyperosmotic conditions significantly reduced SOX9 mRNA in normal P2 and OA P0 but not normal P0 chondrocytes. SOX9 mRNA was stabilised by hyperosmotic conditions. Cyclical loading of normal P2 and OA P0 but not normal P0 cells led to an increase in SOX9 gene expression and this was prevented by MEK1/2 inhibition.

**Conclusions:**

The response to osmotic loading of SOX9 mRNA is dependent on the nature of the osmotic stimulation and the chondrocyte phenotype. This variation may be important in disease progression.

## Introduction

The surfaces of long bones within diarthrodial joints are lined with articular cartilage, an avascular connective tissue that provides a nearly frictionless bearing surface for transmitting and distributing mechanical loads between the bones of the skeleton^1^. The unique load bearing properties of articular cartilage are dependent upon its structural composition and organisation, particularly the interactions between collagens and proteoglycans (PGs) within the extracellular matrix (ECM)^2^. These matrix macromolecules are turned over by chondrocytes embedded within the cartilage. Chondrocytes utilise physical signals, such as mechanical loading and their osmotic environment^3^, to regulate their metabolic activity. During loading, water is expressed from articular cartilage causing the matrix to deform and the PG and mobile cation concentration increase, exposing the chondrocytes to a hyperosmotic environment. Thus physiologically, load-bearing cartilages experience changes in extracellular ion composition and hence osmotic pressure under cyclic and static loading^4^.

Progressive degeneration of articular cartilage leads to joint pain and dysfunction that is clinically identified as osteoarthritis (OA). In healthy tissue there is equilibrium between matrix deposition and degradation; which is disrupted in OA leading to the progressive loss of important matrix components such as collagens and PGs^5^. In early OA, disruption of the collagen network results in an increase in water content of the tissue and a corresponding decrease in pericellular osmolarity^6^. PG loss in later stage OA further exacerbates osmotic perturbations resulting in a decrease in interstitial osmolarity^7^. Under loading OA chondrocytes are subjected to greater variations in osmolarity than normal chondrocytes due to an increase in the rate and extent of fluid loss in this swollen cartilage^8^.

The effects of osmolarity on chondrocyte ECM synthesis have been examined in a number of studies with differing outcomes. These studies have shown that PG synthesis can be reduced in the presence of hyper-, or hypo-osmotic conditions^9,10^. A reduction in COL2A1 gene expression has also been identified as a consequence of hyperosmotic conditions^11^. A further study demonstrated that the application of dynamic hypo-osmotic conditions caused an upregulation in specific cartilage ECM genes^12^. The mitogen-activated protein kinases (MAPK); extracellular signal-regulated protein kinase (ERK) and p38 mitogen-activated protein kinase (p38 MAPK) have been implicated in the adaptive responses of chondrocytes to hyperosmotic conditions^9^. The MAPK families are a cell signalling transduction pathway which connects extracellular signals to intracellular responses such as gene expression in eukaryotic cells^13^. Both p38 MAPK and Jun N-terminal Kinase (JNK) signalling pathways have previously been implicated in osmotic stress signalling in studies in yeast and many mammalian cells^14,15^.

SOX9 is an essential transcription factor controlling the expression of many cartilage ECM genes including collagen type II^16^ and aggrecan^17^. Campomelic dysplasia, a severe syndrome caused by inadequate cartilage formation during development, is due to a haploinsufficiency of SOX9 and underlines its importance to the chondrocyte phenotype^18^. One of the mechanisms responsible for the control of SOX9 expression in chondrocytes is the p38 MAPK-dependant regulation of its mRNA stability^11^. We recently demonstrated that human articular chondrocytes (HAC) exposed to hyperosmotic culture increased the stability of SOX9 mRNA, a process which was also sensitive to p38 MAPK inhibition^11^.

There is no data that determines the effect of hyperosmotic loading on normal vs OA chondrocytes, which are known to undergo changes in phenotype. This study compares the response to hyperosmotic loading of cultured normal and OA primary (P0) chondrocytes. Cell therapy and tissue engineering have the potential to become important treatments in human articular repair but suffer a major limitation, as chondrocytes *in vitro* lose the differentiated phenotype. Thus we wished to identify whether the altered phenotype demonstrated in passaged (P2) chondrocytes effects SOX9 gene expression during hyperosmotic loading. The response of articular cartilage to loading is a complex. Therefore models in which individual physical phenomena can be studied separately are important in determining the cellular mechanisms of joint loading. Our present study was performed to explore the effects of osmolarity on SOX9 expression and the biosynthetic response, by examining whether the activation of the ERK signalling pathways were required. As data from compressive loading experiments clearly indicate that dynamic compression of cartilage produces increases in ECM synthesis by chondrocytes^19^ we also examined the nature of the osmotic load applied to cells.

## Method

### Chondrocyte isolation, expansion and culture

Equine articular cartilage was obtained from the surfaces of metacarpophalangeal joints of skeletally mature horses with grossly normal or arthritic joints. OA joints were derived from clinically diagnosed cases following euthanasia, which on gross pathological inspection exhibited patterns typical of late OA including cartilage fibrillation and erosion, and had disease diagnosed pre-mortem to that joint using clinical methods. Sample collection was subject to institutional ethical review. Isolation of articular chondrocytes has been described previously^20^. The equine articular chondrocytes (EAC) (*n* = 3) were grown as monolayers in Dulbecco’s modified eagles medium (DMEM) (Invitrogen, Paisley, UK), supplemented with 10% foetal calf serum (FCS), 100 units/ml penicillin, 100 μg/ml streptomycin (all from Invitrogen, Paisley, UK) and 500 ng/ml amphotericin B (BioWhittaker, Lonza, USA). Experiments were undertaken with either freshly isolated EAC plated at 100,000 cells/cm^2^ and used within 48 h, or with EAC plated at 20,000 cells/cm^2^ and grown through two passages (with a 1:2 split ratio). In order to elucidate the effects of medium osmolarity on the cells they were grown for 5 h in serum-free and antibiotic-free DMEM adjusted with NaCl to yield 380mOsm or 550mOsm solutions. Previously we confirmed that the effects on SOX9 mRNA were due to the osmotic environment and not caused by increased sodium levels^11^. Following production of the defined media a freezing point depression osmometer (Loser, Berlin, Germany) was used in order to confirm, the osmolarity was within an acceptable range of ±2% variation. Osmolarities used were based upon previous experiments which examined the effects of osmolarity on chondrocytes where 380mOsm control conditions are close to those experienced by healthy chondrocytes *in-situ*, and 550mOsm represented a hyperosmotic condition^9,11,27^. We have recently shown that actin stress fibre formation impaired the response of passage 2 HAC to hyperosmotic load^11^. Therefore passage 2 EAC cultures were supplemented with 10 μM of the ROCK1/2 inhibitor Y27632 (Calbiochem, Nottingham, UK). Where necessary, cultures were also cultured with 10 μM of the MEK1/2 inhibitor U0126 (Sigma-Aldrich, Dorset, UK), for 2 h prior to the commencement of experiments.

### Gene expression analysis

Total RNA was prepared from monolayer cultures in 12-well culture plates using 0.5 ml Tri Reagent (Ambion, Warrington, UK) per well. The Guanidinium thiocyanate–phenol–chloroform extraction technique was used as previously described^21^. M-MLV reverse transcriptase and random hexamer oligonucleotides were used to synthesize cDNA from RNA (both from Promega, Southampton, UK) in a 25 μl reaction. 1 μl aliquots were amplified by PCR in 20 μl reaction volumes on an ABI 7700 Sequence Detector using either a SYBR Green PCR mastermix or a Taqman mastermix where appropriate (Applied Biosystems, Warrington, UK). The fitness of GAPDH as a valid normalisation factor under different osmolarities has been previously established by us^11^. Relative expression levels were normalised to GAPDH and calculated using the 2^−ΔCt^ method^22^. Primers and probes for equine SOX9 and GAPDH were designed by Applied Biosystems Assays-by-Design and had the following sequences: SOX9 Forward; CGC-CGA-AGC-TCA-GCA-AGA, Reverse; CGC-TTC-TCG-CTC-TCG-TTCA, Probe; CAA-GCT-CTG-GAG-ACT-GC; GAPDH Forward; ACT-GGT-GTC-TTC-ACT-ACC-TTG-GA, Reverse; AGC-AGA-GAT-GAT-GAC-CCT-TTT-GG; Probe; AAG-TGA-GCC-CCA-GCC-TT. For determination of aggrecan and COL2A1, SYBR Green detection was used and primers were obtained from Eurogentec (Seraing, Belgium). The primer sequences for aggrecan were designed in Primer Express (Applied Biosystems) software and were: Forward; AGG-AGC-AGG-AGT-TTG-TCA-ACA; Reverse; CCC-TTC-GAT-GGT-CCT-GCT-AT. The primer sequence for COL2A1 and for the housekeeping gene GAPDH have been previously reported^23^. All primers used were designed across exon boundaries.

### RNA decay analysis

For decay experiments freshly isolated EAC (*n* = 4) were grown in monolayers and treated under experimental conditions for 2 h before the addition of 1 μM of the transcription inhibitor actinomycin D (Sigma-Aldrich, Dorset, UK). Decay of SOX9 was then measured following extraction of purified total mRNA at a number of time points 0–3 h later. This was reverse transcribed and real-time PCR undertaken; decay curves were generated using GAPDH as a normalisation factor. Data was plotted on semi-log charts and exponential regression lines generated in Microsoft Excel. The slope (*m*) of the regression lines were used to calculate the mRNA half-life (*t*_1/2_) using the equation *t*_1/2_ = ln(2)/m in order to examine stability.

### Quantification of PG

Glycosaminoglycans (GAG) synthesis was quantified by measuring the incorporation of radioactive ^35^S sulphate. Cartilage explants were from skeletally mature grossly normal metacarpophalangeal joints of horses. Full thickness cartilage was excised and cut into 3 mm diameter explants from the entire surface of the proximal phalanx (*n* = 3). The explants were blotted on sterile gauze pads and their wet weight was recorded. They were then transferred into DMEM supplemented with 10% FCS, 100 units/ml penicillin, 100 μg/ml streptomycin and 500 ng/ml amphotericin B and maintained in 12-well culture plates for 48 h at 37°C in a 5% CO_2_ incubator, to allow synthetic activity to reach equilibrium after harvesting^24^. To evaluate the effect of osmotic loading and MEK–ERK signalling on *de novo* GAG synthesis cartilage explants were labelled in DMEM adjusted to 380 or 550mOsm with NaCl, containing 2 μCi/μl of ^35^S sulphate (MP Biomedicals Inc, Irvine, USA) and, where appropriate, with the MEK1/2 inhibitor U0126 (10 μM). Labelling was performed over 24 h. Sulphate incorporation was determined following papain digestion of the explants^25^. Unincorporated radiolabel was separated from macromolecular products in all samples using PD-10 size exclusion columns (GE Healthcare Lifesciences, Amersham, UK) and eluted in phosphate-buffered saline (Sigma-Aldrich, Dorset, UK)^26^. The ^35^S sulphate radioactivity was measured by liquid-scintillation counting (1410 liquid-scintillation counter; Wallac Oy, Finland) of aliquots from void volume fractions. Total sulphate incorporation rate was calculated for the ^35^S sulphate incorporation rate and normalised to wet weight.

### Statistical analysis

Following normality testing statistically significant differences for *t*_1/2_ data, GAG synthesis and gene expression values of control and treated cultures were analysed using mixed effects linear regression to allow for donors with significant biological variation. Where data are represented as mean values a 95% confidence interval (CI) is used. The analyses were undertaken using S-Plus, SPSS, Minitab and Excel software.

## Results

### Effect of hyperosmolar loading on ECM synthesis

We were interested in the effect of medium osmolarity on ECM production and whether MEK–ERK signalling had a role. Therefore we examined *de novo* GAG synthesis by ^35^S sulphate incorporation in equine cartilage explants cultures. Following 24-h culture at 550mOsm there was an increase in GAG synthesis compared to 380mOsm (19%) but this did not reach statistical significance. Interestingly the presence of the MEK1/2 inhibitor U0126 significantly reduced the incorporation of ^35^S sulphate in both osmotic conditions (*P*=0.04). There was a trend for a multivariable relationship and an interaction between osmolarity and U0126 (*P* = 0.06, *P* = 0.09 respectively). These results suggest that MEK–ERK signalling increases GAG synthesis.

### Matrix gene expression in isolated cells

**Fig. 1:**
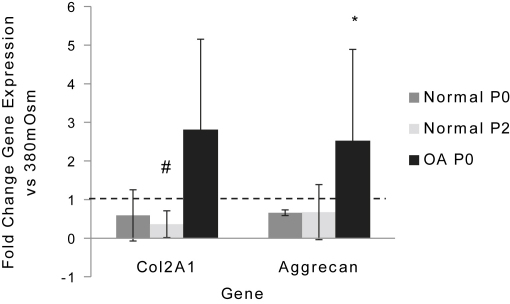
Effect of static hyperosmotic loading on matrix gene expression. Col2A1 and aggrecan mRNA levels in monolayer culture of freshly isolated normal (P0), normal P2 and OA P0 EAC which were incubated at 380 or 550mOsm for 5 h. Data are represented as fold change in expression compared to cells under 380mOsm. Histograms represent means ± 95% CI. # *P* < 0.01, * *P* < 0.05 compared to 380mOsm (represented by dashed line). Data were evaluated using mixed effect linear regression (*n* = 3, three technical replicates per observation).

Next, to define further the downstream effects of static hyperosmolar loading on normal and OA chondrocytes in monolayer culture, we investigated the expression of the cartilage matrix genes COL2A1 and aggrecan, downstream targets of SOX9. Overall we demonstrated a small but significant effect of hyperosmotic conditions on the expression of these genes in dedifferentiated chondrocytes. There was a reduction in COL2A1 mRNA in normal P2 chondrocytes (3 fold ±0.3 SD, *P* = 0.045) whilst OA P0 chondrocytes exhibited an increase in aggrecan mRNA (3 fold ±1.7 SD, *P*=0.05) (Fig. 1).

### Effect of actin stress fibres on SOX9 gene expression in P2 EAC

**Fig. 2:**
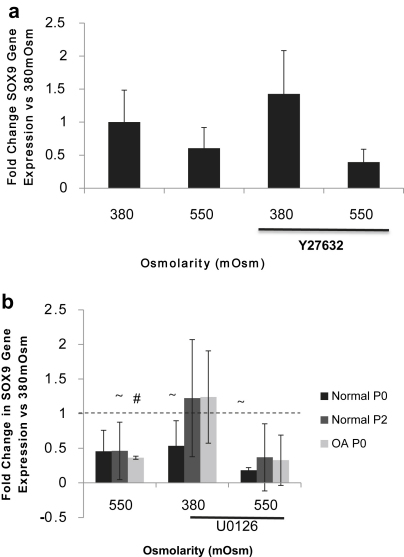
(a) Effect of the ROCK inhibitor Y27632 on SOX9 gene expression in passage 2 EAC. Real-time PCR analysis of SOX9 mRNA in monolayer cultures of cultures exposed to 380mOsm or 550mOsm for 5 h in the absence or presence of the ROCK1/2 inhibitor Y27632. Data are represented as fold change in expression compared to cells under 380mOsm with no inhibitor. Histograms represent means ± 95% CI. Data were evaluated using mixed effect linear regression (*n* = 3, three technical replicates per observation) and no statistical significance was evident in any condition. (b) Effect of static hyperosmotic loading and the MEK1/2 inhibitor U0126 on SOX9 mRNA levels in EAC. Freshly isolated normal P0, normal P2 and OA P0 were chondrocytes cultured at 380 or 550mOsm in the presence or absence of the MEK1/2 inhibitor U0126 for 5 h. Data are represented as the fold change in expression compared to 380mOsm cultures without U0126 (represented by the dashed line). Histograms represent means ± 95% CI. # *P* = 0.0096, ∼ *P* < 0.001 compared to 380mOsm control. (*n* = 3, three technical replicates per observation, mixed effects linear regression).

Previously we identified an inhibitory role for actin stress fibres during hyperosmotic induction of SOX9 in HAC. To examine this further in EAC monolayer cultures of passage 2 EAC, derived from healthy cartilage were incubated in 380mOsm or 550mOsm media for 5 h in the presence or absence of the ROCK1/2 inhibitor Y27632 (10 μM). Analysis of SOX9 mRNA levels in these cultures showed that the ROCK inhibitor Y27632 had no significant effect in cultures exposed to a 5-h period of static hyperosmolar loading [Fig. 2(a)]. Therefore the healthy P2 EAC demonstrated different characteristics to the OA HAC previously examined.

### Effect of hyperosmotic loading on SOX9 gene expression

Freshly isolated EAC from normal or OA joints or passage 2 EAC from normal joints were exposed to static hyperosmolar loading of 550mOsm for a 5-h period in order to determine the effect of hyperosmotic stress on SOX9 mRNA expression. Culture media with an osmolarity of 380mOsm was used as the control condition. Although a 50% reduction in SOX9 mRNA in normal P0 chondrocytes subjected to static hyperosmolar loading was evident [Fig. 2(b)]; this reduction was not statistically significant. In contrast, in P2 normal and P0 OA chondrocytes static hyperosmotic loading significantly reduced SOX9 mRNA [Fig. 2(b)] (65% *P* = 0.0004, 55% *P* = 0.0096).

### Examining the role of ERK in hyperosmotic loading

**Fig. 3:**
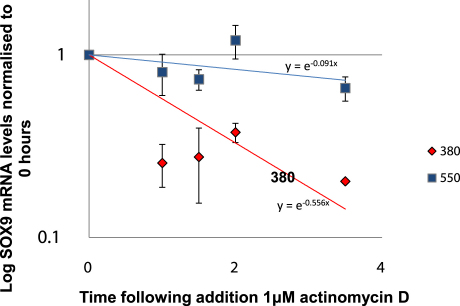
Actinomycin D chase analysis to examine decay of SOX9 mRNA in freshly isolated normal EAC. RNA decay curves are presented for cells cultured at 380mOsm alone or 550mOsm. Data represents the means and 95% CI of the fold changes in SOX9 levels compared to time point zero for four donors, three technical replicates per observation. Slopes of the lines are shown on the graph.

We wished to ascertain whether ERK signalling had an effect on SOX9 gene expression in EAC exposed to hyperosmotic loading. In normal P0 EAC, MEK1/2 inhibition with U0126 significantly reduced SOX9 mRNA under both normosmolar and hyperosmolar conditions (50% and 80% respectively, *P*=0.0003) [Fig. 2(b)]. This reduction was not seen in P2 normal chondrocytes or P0 OA EAC. Previously we found in HAC derived from OA tissue that hyperosmotic conditions led to an increase in the half-life of SOX9 mRNA^11^. Decay curves generated using mean values for all donors identified that culture of normal P0 EAC in 550mOsm produced a trend towards an increase in the stability of SOX9 mRNA (*P* = 0.07) (Fig. 3). Further analysis of the SOX9 mRNA levels at the end of the decay study (3.5 h) revealed a significant increase in SOX9 levels at 550mOsm (*P* = 0.005). We were interested in whether ERK1/2 affects this process but found that treatment with U0126 had no effect on the stability of SOX9 mRNA at 550mOsm (data not shown).

### Effect of cyclical hyperosmotic loading on SOX9 expression

**Fig. 4:**
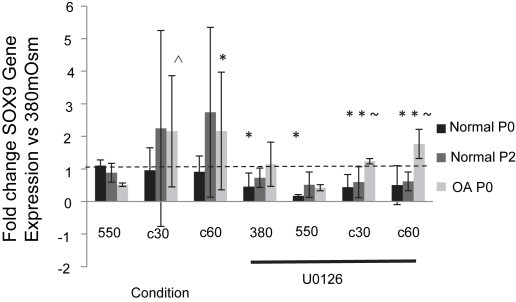
Effect of cyclical hyperosmotic loading on SOX9 mRNA in EAC. SOX9 mRNA levels in monolayer culture of freshly isolate P0, P2 normal and OA P0 EAC incubated under constant 380mOsm, constant 550mOsm or with cyclical application of 380mOsm and 550mOsm every 30 (c30) or 60 (c60) min for the 5-h period. Experiments were carried out in the presence or absence of the MEK1/2 inhibitor U0126 (10 μM). Data are represented as the fold change in expression compared to cells under 380mOsm conditions and without the inhibitor (represented by the dashed line). Histograms represent means ± 95% CI. ^ *P* < 0.07, *P < 0.05, # *P* < 0.01, ∼ *P* < 0.001 (*n* = 3, mixed effects linear regression, three technical replicates per observation).

*In vivo*, chondrocytes subjected to periods of cyclical loading reveal fluid-flow and osmotic fluctuations within the tissue^4^. Therefore we examined the effect of a cyclical application of hyperosmolarity on EAC. Media was changed from 380mOsm to 550mOsm, alternating every 30 (c30) or 60 (c60) min over 5 h. At each frequency the final incubation period was under 550mOsm. In P0 normal chondrocytes dynamic hyperosmolar loading had no affect on SOX9 mRNA. However in normal P2 and OA P0 there was an increase in SOX9 mRNA of 2–3 folds. This was only statistically significant for OA P0 chondrocytes when the culture media was changed every 60 min (*P* = 0.017) though a trend was evident for OA P0 at changes every 30 min (*P* = 0.07) (Fig. 4). We also determined the effects of ERK inhibition on EAC cultured under cyclical hyperosmotic condition (Fig. 4). The presence of MEK1/2 inhibitors in cultures of both normal P2 and OA P0 chondrocytes prevented the increase in SOX9 mRNA caused by both the 30 and 60-min cyclical loading regimes (normal P2 chondrocytes U0126; 77%; *P* = 0.001, and OA P0 chondrocytes U0126; 50%; *P* = 0.044), (Fig. 4). These findings indicate that the MEK–ERK signalling pathway is necessary for the elevation of SOX9 mRNA evident in cyclic hyperosmotic loading of normal P2 and OA P0 chondrocytes. Finally, COL2A1 and aggrecan gene expression were also investigated during cyclic hyperosmotic loading experiments. No change in the expression of these genes was evident in any of the cells or experimental conditions (data not shown).

## Discussion

In chondrocytes some of the changes in matrix expression in response to osmolarity have been well studied^9,27^ however, less is known about these changes in OA chondrocytes. The expression of SOX9 is essential for the ability of the chondrocyte to produce a cartilage matrix^28^ and so we were interested in investigating the expression of SOX9 in normal and OA chondrocytes under osmotic loading, which has been previously un-documented. Given the importance of SOX9 in the development and maintenance of the chondrocyte phenotype, its reduction in OA^28,29^ may contribute to the cartilage pathology. Mechanical stimulation of chondrocytes induces numerous physicochemical changes including alterations in osmotic pressure^30^. This produces a number of physiological and biochemical responses resulting in changes in expression of matrix genes. The nature of the response, in part, depends on the nature of the mechanical stimulation. In general, dynamic stimuli results in an anabolic response, whereas static compression more frequently inhibits chondrocyte activity^31^. The response of chondrocytes from OA cartilage is significantly different from that of normal chondrocytes^32^ suggesting that altered sensing of the osmotic environment and inappropriate responses of the resident chondrocyte population may be important in disease progression.

Previously static hyperosmotic loading for 5 h of P2 HAC derived from OA joints of patients undergoing total knee arthroplasty resulted in an increase in SOX9 mRNA^11^. Contrastingly, here we were intrigued to find that in EAC there was a reduction in SOX9 mRNA although this was only significant in normal P2 and OA P0. During expansion of normal chondrocytes and in OA chondrocytes there is a loss of the specific chondrocytic phenotype and a reversion to a more fibroblast-like phenotype^33–35^. This process appears to alter the response of chondrocytes to a change in osmolarity. However, similar to HAC there was also an increase in the stability of SOX9 mRNA in hyperosmotic conditions^11^. This latter finding would suggest that more SOX9 mRNA should be present. In yeast hyperosmotic stress represses the transcription of the glucose transporter genesHXT2 and HXT4, although there is an increase in transcript half-life^36^ and the effect evident here may be similar resulting in a reduction in the transcription of SOX9 in EAC under hyperosmotic loading. Further studies using nuclear run-on assays^37^, would need to be undertaken to investigate whether this is indeed the case. Interestingly there is an increase in SOX9 during cyclic hyperosmotic loading in normal P2 and OA P0 but not normal P0 chondrocytes, which is in agreement with our findings in HAC OA P2 chondrocytes (unpublished data). These data suggest that there is only an effect on SOX9 mRNA amounts through both static and cyclic hyperosmotic loading on chondrocytes with altered phenotype, from either culture dedifferentiation or phenotypic alteration from disease in equine tissue. Modifications of the articular chondrocyte phenotype are commonly observed in OA cartilage, including suppression of genes involved in the phenotypic stability of articular chondrocytes^38^, reduced ECM protein production^39^, proliferation and change in morphology^40^. During expansion of normal chondrocytes and in OA chondrocytes there is a loss of chondrocytic phenotype and a reversion to a more fibroblast-like phenotype^35^. This phenotypical change is accompanied by decreased gene expression of cartilage specific markers like COL2A1 and aggrecan. This process could also alter the response of chondrocytes to extracellular stimuli such as a change in osmolarity. In normal HAC cyclic stretch has an anabolic effect as shown by increases in aggrecan expression. However, this effect was not evident in OA chondrocytes, where no change in the expression of either gene was observed^41^. This difference might be attributed to a change in mechanotransduction pathways between normal and OA chondrocytes^42^.

A previous study undertaken in bovine chondrocytes encapsulated in alginate beads for 48 h indicated an increase in sulphate incorporation in response to hyperosmotic conditions which was abrogated in the presence of the MEK inhibitor PD98059^9^. Here we demonstrate that MEK–ERK signalling effects GAG synthesis in normal equine cartilage explants and there is a trend for an interaction between osmolarity and MEK–ERK signalling. We examined aggrecan and COL2A1 levels in our cultures and found that hyperosmolarity had little effect on their expression in most experimental conditions. However, there was a reduction in COL2A1 in normal P2 chondrocytes agreeing with the majority of studies that have noted a decrease in ECM production under static hyperosmotic loading^31^. Intriguingly there was an increase during static hyperosmolar loading in aggrecan mRNA in OA P0 chondrocytes, similar effects have been revealed in human and bovine intervertebral disc cells exposed to hyperosmotic conditions^43^. This is in contrast to other studies in normal chondrocytes where an increase in osmolarity results in a reduction in aggrecan mRNA^27^.

Many cells exist in an environment where osmolarity can fluctuate and have a variety of responses; these often appear to be controlled through a signalling network of protein kinases and transcription factors. In mammalian cells hyperosmolarity activates many MAP kinases including ERK1/2 MAPK. In yeast cells although ERK activity is not essential for the transcriptional regulation of BGT1 and SMIT, genes that encode for osmolyte transporters^44^, inhibition of MEK1 down regulated TonE-mediated reporter gene expression^45^ and it has been proposed that the activation of ERK pathway in hyperosmotically stressed cells serves as a cell survival signal^46^. Interestingly studies on rat nucleus pulposus cells, which produce an ECM similar to that of chondrocytes, indicated exposure to a hyperosmotic environment resulted in an increase in the transcription factor TonEBP with a subsequent activation of its target genes including aggrecan. This transactivation was sensitive to inhibition of ERK signalling^47^. Others have demonstrated that MEK–ERK signalling is activated in articular chondrocytes experiencing normal osmotic conditions, exposed to fluid-flow leading to a down-regulation of aggrecan^48^. In the present study the elevated response of SOX9 mRNA under cyclic hyperosmotic loading is dependent on MEK–ERK signalling which is comparable to findings in HAC (unpublished data). Although MEK–ERK signalling was not required for the reduction in SOX9 apparent in static hyperosmotic loading of normal P2 and OA P0 equine chondrocytes, in normal P0 chondrocytes blocking this pathway using the MEK1/2 inhibitor U0126 demonstrated that MEK–ERK signalling represses SOX9 expression in normal chondrocytes. Hyperosmotic stress has been previously shown to activate ERK in tissue culture^49^. Furthermore in young murine P0 chondrocytes there was an increased Sox9 expression, caused by FGF-2 stimulation which was inhibited by U0126^50^. These results demonstrate a similarity between the reactions of young murine costal chondrocytes and normal equine chondrocytes which were used in this study.

From these studies we have found that the nature of the response to osmotic loading of SOX9 mRNA is dependent on the nature of the osmotic stimulation and on the chondrocyte phenotype. The response of chondrocytes from OA cartilage is significantly different from that of normal chondrocytes suggesting that altering sensing of the osmotic environment and inappropriate responses of the resident cell population may be important in disease progression.

## Authors’ contributions

MP participated in the design of the study and the experiments as well as performing the majority of the experiments, statistical analysis and interpretation of the data. ST and PM participated in the design of the study and the interpretation of the data. PC participated in the design of the study and the experiments, some of the statistical analysis and supervised all the experiments. All authors read and approved the final manuscript.

## Role of the funding source

This study was supported by a Wellcome Trust Veterinary Research Entry Level Fellowship to MJP (WT085324MA). SRT is supported by Arthritis Research UK.

## Conflict of interest

The authors have no conflicts of interest to report.
